# Coordinated, Long-Range, Solid Substrate Movement of the Purple Photosynthetic Bacterium *Rhodobacter capsulatus*

**DOI:** 10.1371/journal.pone.0019646

**Published:** 2011-05-04

**Authors:** Kristopher John Shelswell, J. Thomas Beatty

**Affiliations:** Department of Microbiology and Immunology, University of British Columbia, Vancouver, British Columbia, Canada; National Institutes of Health, United States of America

## Abstract

The long-range movement of *Rhodobacter capsulatus* cells in the glass-agar interstitial region of borosilicate Petri plates was found to be due to a subset of the cells inoculated into plates. The macroscopic appearance of plates indicated that a small group of cells moved in a coordinated manner to form a visible satellite cluster of cells. Satellite clusters were initially separated from the point of inoculation by the absence of visible cell density, but after 20 to 24 hours this space was colonized by cells apparently shed from a group of cells moving away from the point of inoculation. Cell movements consisted of flagellum-independent and flagellum-dependent motility contributions. Flagellum-independent movement occurred at an early stage, such that satellite clusters formed after 12 to 24 hours. Subsequently, after 24 to 32 hours, a flagellum-dependent dispersal of cells became visible, extending laterally outward from a line of flagellum-independent motility. These modes of taxis were found in several environmental isolates and in a variety of mutants, including a strain deficient in the production of the *R. capsulatus* acyl-homoserine lactone quorum-sensing signal. Although there was great variability in the direction of movement in illuminated plates, cells were predisposed to move toward broad spectrum white light. This predisposition was increased by the use of square plates, and a statistical analysis indicated that *R. capsulatus* is capable of genuine phototaxis. Therefore, the variability in the direction of cell movement was attributed to optical effects on light waves passing through the plate material and agar medium.

## Introduction

Cells may respond differently to factors such as nutrients, temperature, and light [1], and motility is a fundamental response that allows bacteria to respond to their environment. Motility provides bacteria with a means of escaping detrimental surroundings and moving toward conditions that are favourable for growth [2]. Bacterial motility occurs in both aqueous [3], [4] and non-aqueous environments [5], but no single type of movement appears to be best for all conditions. Non-aqueous, or solid-substrate, motility has been recognized in a growing number of bacterial species and several motility mechanisms have been identified, including swarming, twitching, sliding, and gliding motility [6].

Swarming motility is driven by flagellar rotation in a film of fluid on the surface of the substrate [7]. Cells are typically hyperflagellated and secrete surfactive compounds that increase the fluidity on the substrate over which the cells are moving [8], [9].

Twitching motility is mediated by the polymerization and depolymerization of long polar pili [10]. Retraction of the extended pilus at the cell envelope pulls the cell forward toward the distal tip of the pilus that is anchored to the substrate [11].

Sliding motility is a passive mechanism that occurs on moist surfaces in the absence of flagella and pili [5], where the expansive force of cell proliferation moves cells at the periphery of a cell mass. The peripheral cells move outward when the force of the cell mass exceeds the adhesion between cells and the substrate, and cells may secrete surfactant compounds that decrease the surface tension on the substrate [12], [13].

Gliding motility occurs without flagella or pili, although unlike sliding it is an active form of movement. The linear movements of gliding cells may consist of smooth, continuous translocations or sporadic advancements [14], which appear to be achieved by at least three separate mechanisms. Rearrangements in the shape of the cell that generate standing waves, the secretion of material from the poles or girdle of cells, and localized adhesions along the cell surface have been proposed as mechanisms that propel bacterial gliding motility [15], [16].

Although cells of some species can move individually on surfaces, cells often cluster together and align into ordered masses that move together. Swarming cells form motile rafts [17], twitching cells break out into spearheads [18], and sliding motility requires groups of cells to generate the expansive force that moves the periphery outward [5]. Gliding movements have been reported as individual cells, as in the adventurous movement of *M. xanthus* [19], or as aggregated rafts [20]. In general, a coordinated aggregation of cells appears to facilitate solid-substrate bacterial movement.

*R. capsulatus* swims using a polar flagellum in aqueous conditions [21], but flagellar swarming on solid surfaces has not, to our knowledge, been shown in this bacterium. We have previously reported flagellum-dependent and flagellum-independent *R. capsulatus* motility in the interstice between an agar medium and a borosilicate Petri plate [22]. Flagellum-dependent motility in such an interstice is thought to be a form of swimming movement aided a thin interstitial film of water between the two substrates [23], because the diffuse flagellar pattern is not observed on the agar surface of an agar-air interface. Flagellum-independent motility is thought to occur on the surface of the agar medium in the interstice, as flagellum-independent motility also occurs on the agar surface of an agar-air interface. In this paper we describe characteristics of flagellum-independent and flagellum-dependent motility in the interstice between an agar medium and a borosilicate glass surface. We show that individual cells are capable of movement, but that long-range movement toward white light occurs as a coordinated group of cells independently of the flagellum. Although the mechanism driving flagellum-independent motility in *R. capsulatus* is unknown, the available evidence indicates that movement may be mediated by gliding motility.

## Materials and Methods

### Bacterial strains and growth conditions

The *R. capsulatus* strains are described in Table 1. Strain B10 [24] was used as the parental strain for mutant construction, and the source of DNA for genetic manipulations. A defined minimal medium (RCV) containing malate as the carbon source and ammonium as the nitrogen source [25] was used for aerobic aqueous cultures, whereas a complex medium (YPS) containing yeast extract and peptone [26] was used for photosynthetic aqueous and solid-substrate cultures.

**Table 1:** *R. capsulatus* strains.

Strains	Markers and Relevant Properties	Source or Reference
B6	environmental isolate from St. Louis, MO pond sample; no GTA production	[24]
B10	environmental isolate from St. Louis, MO pond sample; wild type	[24]
BCKF	*ctrA*::KIXX, derived from strain B10; no flagellum	[21]
bKSDF	Δ*flaA*, derived from strain B10; no flagellum	this work
bKSDFgtaI	Δ*flaA*, Δ*gtaI*, derived from strain BLKI; no flagellum or quorum sensing	this work
SP36	environmental isolate from Bloomington, IN sewage settling pond	[26]
YW1	environmental isolate from Yellowwood State Forest, IN	[26]
YW2	environmental isolate from Yellowwood State Forest, IN	[26]

Culture turbidity was monitored by measuring light-scattering with a Klett-Summerson photometer (red filter #66). One hundred Klett units are equivalent to approximately 3×10^8^ colony-forming units per mL.

### Motility assays

The standard solid-substrate motility assay utilized a stab inoculation of 20 mL YPS agar (1.5% w/v) in a borosilicate Petri plate. Cells from an exponential phase aerobic culture were collected by centrifugation and the resulting cell pellets were sampled using a sterile square toothpick, to inoculate the plate-agar interstice. Plates were incubated in a motility chamber under aerobic conditions with broad spectrum white light tungsten filament lamp (Sylvania, 60 W) illumination at 30°C [22]. The light source consisted of an array of 4 unfocused lamps arranged at the corners of a 12.5 by 13.5 cm^2^ rectangular area. The light source was positioned 14 cm away from the edge of plates, which were stacked in two vertical columns with eight plates each. In some experiments square polystyrene plates were used, but because motility does not occur in the agar-polystyrene interstice, a glass microscope slide was aseptically added to square plates to provide a glass-agar interstice for cell movement.

### Statistical measurement of the direction of cell movement

**Figure 1:**
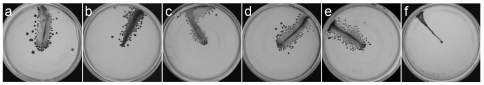
Representative agar plates inoculated with *R. capsulatus* environmental isolate strains, and incubated at 30°C aerobically for 48 hrs with white light illumination. (a) B6; (b) B10; (c) SP36; (d) YW1; (e) YW2; (f) bKSDF. Illumination was from the top of the images.

Although there appeared to be a predisposition for cells to move in the general direction of the light source, there was great variability in different experiments (Figure 1). Cell movement was analyzed using a 2-D geometrical evaluation, to measure the direction of cell movement after 48 hours of incubation. The direction of cell movement corresponded to the farthest point of visible cell density from the point of stab inoculation. Directional motility was measured as an angular value between the direction of cell taxis and a fixed reference point at the edge of the Petri plate. The reference point, designated as 0°, corresponded to the plate position closest to the source of illumination. The 0° reference point is positioned at the top of plates presented in figures throughout this publication. The radius of cell density at the air-medium interface varied from approximately 1 to 4 mm, so samples moving less than 4 mm from the point of stab inoculation were considered to not move. Statistical analysis was performed assuming an outlier data cutoff derived from multiples of the interquartile range (IQR), and the non-parametric two-independent sample Wilcoxon (P_Z_) test, which does not assume normally distributed data. The IQR model, measured outward from the median, is based on standard deviations derived from a normal Gaussian distribution [27]. The Wilcoxon test assigns a rank to each data point and compares the ranked sum of the distributions [28]. P_Z_ values were determined for the 0.95 confidence interval to represent differences between samples that were not the result of random chance. P_Z_ values of >0.05 were considered to represent no statistically significant difference in the distance or direction of motility. P_Z_ values of <0.05 were considered to represent statistically significant differences in the distance or direction of motility.

### Time-course observations of cell movement in plates

Stab-plates were observed and recorded at regular intervals in order to follow cell movements. Plates were removed, photographed, and replaced in the motility assay chamber in the same orientation at 0, 0.5, 1, 2, 4 hours after stab inoculation, and subsequently at 4-hour intervals up to 48 hours following inoculation. The ^32^P-orthophosphate-labelling of cells was done as described (Shelswell et al), and a stab-plate of labelled cells was incubated for 48 hours before being photographed, and used to expose X-ray film to capture an autoradiograph.

### Light microscopy

Glass bottom plastic plates (50×7 mm circular dish with 40 mm glass insert, Willco Wells B.V., Amsterdam) were examined in the light microscope after stab-inoculation and incubation with broad spectrum white light tungsten filament bulb illumination at 30°C for 24 hours. The microscopy conditions were under oil immersion with differential interference contrast/Nomarski interference contrast (DIC/NIC), using a Zeiss 510 Meta scan head mounted on an Axiovert 200 M inverted microscope (100× magnification) at room temperature. Images were recorded over a period of 180 minutes using a Zeiss AxioCam HRm CCD camera controlled by Zeiss LSM Software. Images were edited and converted to JPEG images using Axio Vision LE version 4.5 (Carl Zeiss Canada Ltd., Toronto, ON).

## Results

### Solid-substrate motility is a conserved phenotype in independently isolated *R. capsulatus* strains

The occurrence of solid-substrate motility in *R. capsulatus* was tested in five strains isolated from three different locations. The plate-medium interstice was inoculated by stabbing the center of plates, which were incubated for 48 hours. All strains were capable of movement away from the point of inoculation in the plate-medium interstice. As shown in Figure 1, the motility of strains B6 [24], SP36, YW1, and YW2 [26] closely resembled that as previously described for the wild type strain B10 [22]. All five environmental isolate strains exhibited two patterns of movement: 1) the broad, diffuse movement pattern due to flagellum-dependent contributions; and 2) the dense, linear flagellum-independent pattern of movement. This is in contrast the *flaA* mutant strain bKSDF that lacks the flagellum, and which exhibited only the dense linear motility pattern of flagellum-independent contributions (Figure 1f) as previously described [22]. The experiments described below were to further dissect this pattern of motility, using the wild type strain B10 and mutant derivatives.

### Coordinated movement of a subset of cells in the plate-agar interstice

**Figure 2:**
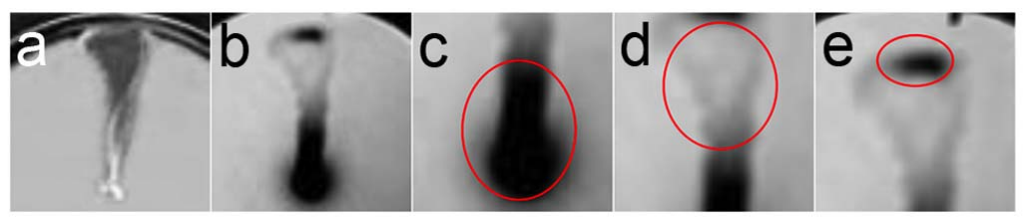
An agar plate inoculated with ^32^P-orthophosphate-labelled *ctrA* mutant strain BCKF. (a) photograph of cell proliferation visible to the eye; (b) autoradiogram showing ^32^P signal profile; (c) high signal intensity near the point of inoculation; (d) low ^32^P signal intensity along taxis path; (e) high ^32^P signal intensity at leading edge of taxis. Illumination was from the top of the images.

In one approach, the distribution of ^32^P-labelled *R. capsulatus ctrA* mutant cells in the plate-medium interstice was examined 48 hours after inoculation. The *ctrA* mutation blocks the production of the flagellum [21], and so the movement of this strain is due to a flagellum-independent mechanism [22]. As shown in Figure 2a, the visible appearance of the plate after 48 hours was that of a more or less continuous line of cell density from the central point of inoculation to the edge of the plate. However, the distribution of ^32^P-labelled cells, as revealed by autoradiography, differed. The autoradiograph indicated that the radioactive signal was greatest at the point of inoculation and extended for only part of the way along the line of cell movement, with a cluster of radioactive cells at the end of the line of cell movement (Figure 2 b–e).

We interpret these results as indicating that most of the cells did not move, or moved a short distance before stopping (Figure 2c). In contrast, the cells at the edge of the plate must have originated from an organized cluster that rapidly translocated from the point of inoculation to the plate perimeter in a coordinated manner, resulting in a strong ^32^P signal (Figure 2e).

We hypothesize that an organized cluster of cells leading the line of movement occasionally shed cells along the way, and that these cells subsequently divided, decreasing the amount of ^32^P per cell (Figure 2d) and filling in the line of growth between the center and the edge of the plate after the leading cluster of cells halted at the edge of the plate. This hypothesis was evaluated in time-course experiments, to see whether cell density appeared at the periphery of plates before filling in the region between the inoculation point and the end of the line of cell movement.

### Coordinated movement of cells in the plate-agar interstice

**Figure 3:**
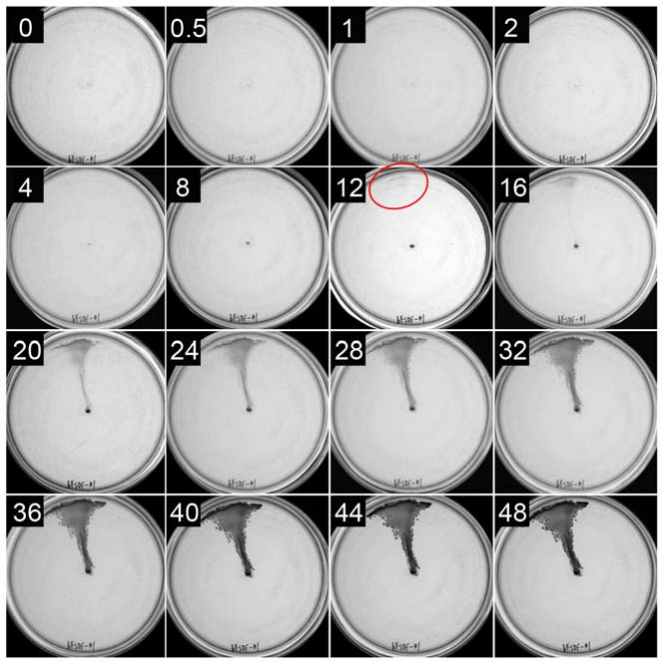
An agar plate inoculated with *flaA* mutant strain bKSDF. The plate was photographed at intervals (0 to 48 hours) after inoculation, as indicated by the number (of hours) in the upper left of each image. The macroscopically visible appearance of the satellite cluster is indicated with a red circle. Illumination was from the top of the images.

The ^32^P labelling experiment provided information regarding cell organization only at the end of 48 hours, and so plates stabbed with several different strains were examined at time intervals after inoculation, up to 48 hours. For comparison to the flagellum-independent motility of the *ctrA* mutant given in Figure 2, a time-course experiment on the flagellin-deficient *flaA* mutant bKSDF is shown in Figure 3. There was little or no change in the appearance of the plate, other than some growth at the stab point at the center of the plate, for 8 hours. However, after 12 hours, a patch of cell density became visible at the edge of the plate. The cell density at the plate edge was stronger after 16 hours, but it was not until the 20 hour time-point that a line of cell density connecting the edge of the plate to the central inoculation point became visible. This line of cell density became stronger and wider over the following time-points, and a few colonies appeared.

We suggest that the time-course experiment shown in Figure 3 supports our interpretation of the data shown in Figure 2. Namely, the flagellum-independent motility of *R. capsulatus* in the plate-agar interstice occurs by rapid movement of a subset of cells from the point of inoculation to the periphery of the plate, giving rise to the cell density seen after 12 hours in Figure 3. As the collection of cells moves, a relatively small number of cells are shed and remain stationary, giving rise to the line of growth not visible until after 20 hours in Figure 3. Cells might have moved farther within this time period, given a glass-agar interstice with a greater distance between the site of inoculation and the edge of the plate. We next describe experiments on the wild type strain B10, and other mutant derivatives, which show that coordinated movement of a mass of cells is not specific to *ctrA* and *flaA* flagellum-deficient mutants.

**Figure 4 pone-0019646-g004:**
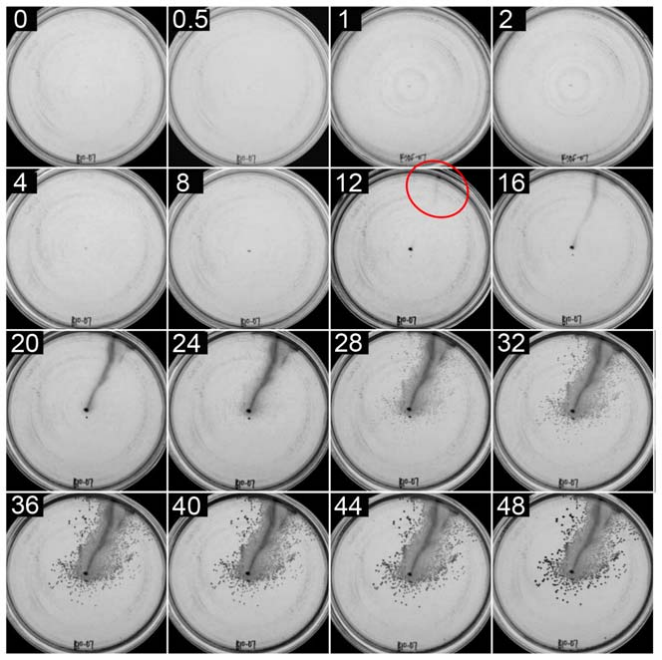
An agar plate inoculated with wild type strain B10. The plate was photographed at intervals (0 to 48 hours) after inoculation, as indicated by the number (of hours) in the upper left of each image. The macroscopically visible appearance of the satellite cluster is indicated with a red circle. Illumination was from the top of the images.

Figure 4 shows a time-course experiment on the wild type strain B10, in which cell density became visible near the edge of the plate 12 hours after inoculation, whereas a clear, continuous line of growth back to the inoculation point was not visible until the 20 hour time point. Consistent with previous work [22], it appears that the flagellum is not needed for motility under these conditions, although the presence of the flagellum increases the lateral dispersion of cells. Furthermore, it appears that a flagellum-independent mechanism predominates in the first 12 to 20 hours after inoculation of a plate, whereas flagellum-dependent movement does not become macroscopically obvious until 24 to 36 hours after inoculation (compare Figures 3 and 4).

Because the experiments described above indicated that a subset of cells moved *en masse* from the point of inoculation to the periphery of the plate, it was possible that a concentration-dependent signal was exchanged between individual cells, as in quorum-sensing [29]. In fact, *R. capsulatus* contains the *gtaI* gene that encodes an enzyme that produces an acylated homoserine lactone that induces the expression of a phage-like gene transfer agent (GTA) [30]. To evaluate whether this quorum-sensing system is needed for rapid long-range cell movement, the *flaA*/*gtaI* knockout strain bKSDFgtaI was evaluated in a time-course experiment.

**Figure 5:**
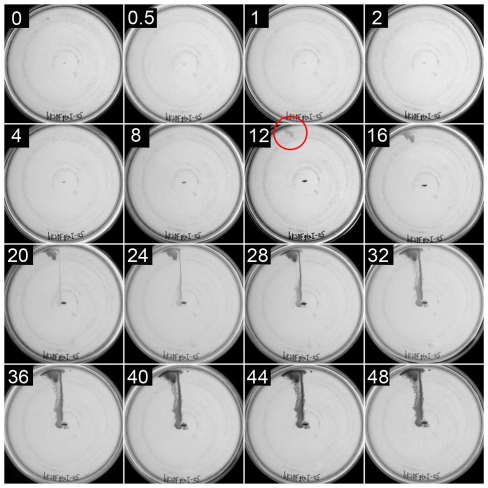
An agar plate inoculated with *flaA*/*gtaI* double mutant strain bKSDFgtaI. The plate was photographed at intervals (0 to 48 hours) after inoculation, as indicated by the number (of hours) in the upper left of each image. The macroscopically visible appearance of the satellite cluster is indicated with a red circle. Illumination was from the top of the images.

As shown in Figure 5, this double mutant yielded growth at the plate periphery after 12 hours in the absence of the GtaI-dependent quorum sensing signal, earlier and more strongly than at the inoculation site at the center of the plate. At later times, beginning at the 20 hour time point, intervening growth between the periphery and the site of inoculation yielded a continuous line of growth as in the experiments on the other strains. Therefore it appears that quorum sensing is not needed for this flagellum-independent movement of cells *en masse*.

The extent of macroscopically visible cell density observed across the plate-medium interstice varied (Figure 1) so we attempted to quantify the contributions from the flagellum-dependent and flagellum-independent motility mechanisms. The width of macroscopically visible cell density was measured at the halfway point between the point of inoculation and the edge of the plate. This midpoint was used to avoid any effects of elasticotaxis from the stab inoculation [31] or spatial crowding from encountering the edge of the plate. The average amount of cell spreading from the path of flagellum-independent motility during time-course experiments is summarized in Table 2. The average measured width of cell density in the wild type strain B10 was greater than 4.5 fold that of bKSDF and 7 fold that of bKSDFgtaI, with a B10 strain standard deviation greater than 3 fold that for both mutant strains (Table 2). The difference between the measured width of *flaA* and *flaA*/*gtaI* movement was significantly lower at approximately 1.4 fold the average and 1.2 fold the standard deviation (Table 2).

**Table 2:** Statistical analysis of the width of macroscopically visible cell density associated with cell movement patterns in the plate-medium interstice.

Strain	Genotype	Average Movement Pattern Width (mm)(a)	P_Z_ (b)	Number of Samples
B10	wild type	44.1 (14.6)		24
bKSDF	*flaA*	9.1 (4.4)	6.9×10^−9^*	24
bKSDFgtaI	*flaA gtaI*	6.2 (3.6)	3.9×10^−9^*	24

(a) Measured at midpoint between the point of inoculation and the edge of plate. Standard deviation in parentheses.

(b) Two-tailed two-independent sample Wilcoxon sum test of significant difference compared to wild type B10 sample pattern width distribution. For a >0.95 probability that the width distribution of movement was different from B10 controls, P_Z_<0.05. Asterisks indicate cases where P_Z_<0.05.

The fluid content of the surface significantly effects solid substrate motility of prokaryotes [5], [32], [33], so perhaps the evaporation of moisture during incubation affected the magnitude of flagellum-dependent contributions. The removal and replacement of plates in the incubation chamber following time point measurements resulted in small changes in the initial orientation with respect to the light source. This could have resulted in the broadening of the line of flagellum-independent cell density. Therefore we believe that the variation in the extent of flagellum-dependent dispersal from the path of flagellum-independent movement was attributed to random differences in experimental conditions such as moisture content in the plate-medium interstice. Furthermore, variation in the width of the flagellum-independent movement pattern may have been attributed to slight fluctuations in the perceived direction of illumination following time-course measurements.

### Microscopic evaluation of the rate of flagellum-independent cell movement in the plate-medium interstice

**Figure 6:**
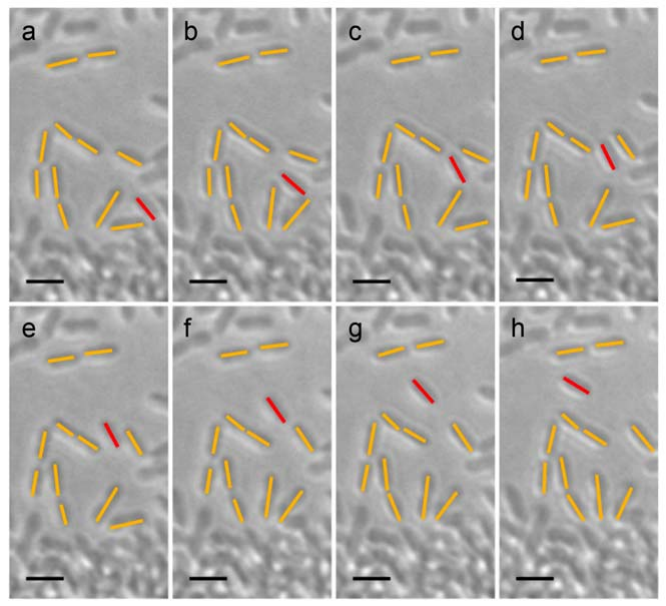
Light microscopy of an agar plate inoculated with *flaA* mutant strain bKSDF. (a–h) long-range cell movement. Red designates the motile cell of interest, orange designates surrounding stationary cells, and the black scale bar represents approximately 5 µm. An image was captured each 0.25 seconds over the course of 100 seconds. Only 8 of the 400 frames are shown from a cumulative 23 second interval of forward cell movement: (a) 8.75 sec; (b) 12.25 sec; (c) 15 sec; (d) 24.5 sec; (e) 29.25 sec; (f) 55.5 sec; (g) 57.5 sec; (h) 91.5 sec.

The edge of confluent growth in the glass-agar interstice of plates 24 hours after inoculation was examined by microscopy of flagellum-deficient strain bKSDF stab-plate samples. Masses of moving cells were not found, and most of the cells were non-motile. However occasionally individual cells were observed to undergo intermittent, discontinuous movements over a distance of several cell lengths (Figure 6). Movements were directed along the long axis of the cell, with occasional reversals in direction, and motility was observed independently of the proximity of other cells (see also Video S1). The motile cell highlighted in Figure 6 moved approximately 25 µm over the course of 100 seconds, at a rate of ∼0.25 µm/sec. However, the cell was observed to move for only 23 of the 100 seconds because movement was punctuated by frequent stops. Thus the actual rate of cell movement, not including stops and reversals, was ∼1.1 µm/sec. These observations showed that individual cells are capable of long-distance movement in the glass-agar interstice independently of the single, polar flagellum produced by wild type *R. capsulatus* strain B10 cells, and perhaps this mechanism is used by a mass of cells moving in a line to the edge of a plate.

### Movement of cells toward white light

**Figure 7:**
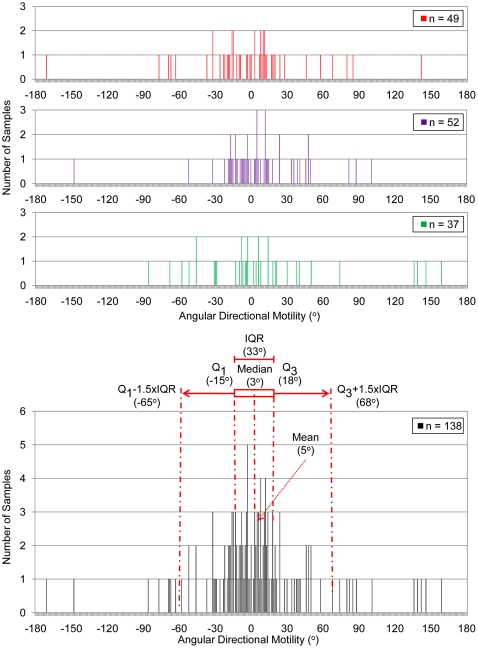
Graphical representation of the measured angular direction of motility of *R. capsulatus*. Cells were inoculated into the glass-agar interstice of square plates with a borosilicate insert, and incubated with incident white light. 0° indicates movement directly toward the light source, and ±180° indicate movement directly away from the light. Q_1_, first quartile; Q_3_, third quartile; IQR, interquartile range; n, total number of samples: (red) wild type strain B10; (purple) *ctrA* mutant strain BCKF; (green) *flaA* mutant strain bKSDF; (black) pooled B10, bKSDF, and BCKF strains with statistical analysis.

Although there appeared to be a general trend for cells to move toward the direction of the light source, there was significant variability in the direction of movement (Figure 1). Therefore a statistical analysis of 138 motility experiments was done. This analysis was based on 49 measurements of the wild type strain B10 [24], 52 measurements of the B10-derived strain BCKF (*ctrA* ^−^) [21], and 37 measurements of the B10-derived strain bKSDF (*flaA* ^−^). The direction of movement was determined by drawing a line from the point of inoculation to the end point of movement, and measuring the angle relative to the direction of the illumination. The distribution of the direction of movement is presented in Figure 7, where the height of each bar represents the number of times that cells travelled in a particular angular direction.

In this statistical analysis, the interquartile range (IQR) was used to identify outliers, data that fall outside the observed trend in the distribution of the data. The IQR analysis of the distribution was similar for both the mutant BCKF and bKSDF strains when compared to the wild type strain B10. Furthermore, a Wilcoxon distribution analysis indicated that there was no statistically significant difference between the distribution of the motility for both BCKF and bKSDF mutant strains when compared to the wild type strain B10 (Table 3). This indicated that the CtrA and FlaA proteins were not required for a directional response to broad spectrum white light. Because the three strains had distributions that did not differ in a statistically significant manner, samples were pooled to provide a more robust statistical population. Relative to the direction of the lamp at 0°, the pooled IQR was between −15° and 18° in 70 (∼51%) of the experiments. The distribution appeared to follow a normal Gaussian curve because there were few outliers, based on the spread of the distribution and the IQR (Figure 7 and Table 3). Outliers are ≥3 standard deviations from the mean, and can be an outside (±1.5 IQR) or a detached (±3 IQR) value, with respective probabilities of 0.05 or 0.005 [27]. The directional movement measured in 120 (87%) of the experiments was within the outlier range between −65° and 68°. These results indicate that *R. capsulatus* cells move toward the direction of white light illumination during incubation in the plate-medium interstice of stab-plate experiments, in what appears to be a genuine phototactic response.

**Table 3:** Statistical analysis of the directional distribution of cell movement and comparison of direction of movement to the wild type strain B10.

Strain	Genotype	Mean	Median	IQR	Q_1_	Q_3_	Outlier Range(a)	P_Z_ (b)	Number of Samples
B10	wild type	−1	0	32	−19	13	−67, 61		49
BCKF	*ctrA*	7	4	33	−11.5	21	−60, 70	0.242	37
bKSDF	*flaA*	11	2	34	−13	21	−64, 72	0.533	52
Pooled(c)		5	3	33	−15	18	−65, 68	0.456	138

Directional values in angular degrees relative to the direction of the light source set at 0° and the point of inoculation (as in Figure 7).

(a) Outlier range determined by interquartile range (IQR) distribution (Q_1_−1.5IQR, Q_3_+1.5IQR).

(b) Two-tailed, two-independent sample Wilcoxon rank sum test of significant difference compared to wild type B10 sample directional distribution. For a >0.95 probability that the directional distribution of movement was different from B10 controls, P_Z_<0.05.

(c) Pooled population consisting of all B10, BCKF and bKSDF strain samples.

## Discussion

### Solid-substrate motility in several *R. capsulatus* isolates from nature

Solid-substrate motility has been documented in an increasing range of prokaryotic species [5], [34], and the results obtained with *R. capsulatus* strains B6, B10, SP36, YW1, and YW2 (Figure 1), suggest that it is a common trait in this species. Furthermore, both the flagellum-dependent and flagellum-independent movement contributions previously described [22] also appear to be common traits in *R. capsulatus*. This is because all the environmental isolate strains that were evaluated yielded a pattern of motility in which the broad, flagellum-dependent motility phenotype was superimposed on a line of cell movement that remains in *ctrA* and *flaA* mutants that lack the flagellum.

### Organization of *R. capsulatus* motile cells, satellite clusters, and coordinated multi-cellular movement

The autoradiogram of ^32^P-labelled *R. capsulatus* cells taken 48 hours after inoculation at the center of a plate indicated that a group of cells from the inoculum moved rapidly from the inoculation site to the edge of the plate (Figure 2). The swarming, twitching, or gliding movement of several prokaryotic species is facilitated by the organization of multicellular rafts or spearheads [17], [35], [36], and so perhaps this is the case also for *R. capsulatus*. To determine whether a subset of the cells inoculated into the center of a plate moved rapidly to the periphery, with subsequent growth of cells left behind along a trail, time-course experiments were done. It was thought that in these experiments it might be possible to see macroscopic growth near the periphery of plates, before there was visible growth in the line connecting the inoculum site to the edge of the plate.

It was found that a mass of *R. capsulatus* cells appeared at the edge of the plate, driven by flagellum-independent movement away from the site of inoculation. These satellite clusters appeared before macroscopically visible cell density connecting the cells to the site of inoculation (Figures 3 through [Fig pone-0019646-g004] 5). We estimate that the number of cells in a newly visible satellite cluster to be on the order of a single pin-point *R. capsulatus* colony visible to the naked eye, approximately 10^6^ cells. If cells doubled every 2.5 hours [37], [38], approximately 50 hours (20 generations) would be required for a single cell to form the visible *R. capsulatus* satellite clusters. However, satellite clusters appeared after as little as 12 hours (∼5 generations). Therefore, the satellite clusters appear to have formed from a group of cells at the site of inoculation that moved as an aggregated mass.

In multiple experiments, satellite clusters became visible within 24 hours, whereas some appeared as early as 12 hours after inoculation. This was observed in all three strains described, and we attribute this to variability in the number of cells initially inoculated into the interstice. The stab-plate inoculation method (using a toothpick dipped into cells pelleted by centrifugation) does not introduce a uniform number of cells into the plate-medium interstice, which may account for the variability in the time needed for the initial appearance of the satellite clusters. A large number of cells travelling in a coordinated mass would require fewer cell divisions to become macroscopically visible, and such a satellite cluster would appear before a sample with a small number of cells inoculated into the plate-medium interstice.

### Temporal differences in contributions from separate motility mechanisms

In the time-course experiments the appearance of plates stabbed with wild type cells after ∼12 to 20 hours resembled the appearance of later stages of plates stabbed with strains lacking the flagellum. Thus, it appears that flagellum-independent motility dominates early in these experiments, whereas flagellar motility dominates at later times. Eventually movement ceases, presumably because of nutrient depletion (Figure 4).

Different species of bacteria exhibit different trends in flagellar gene expression as cultures progress from the exponential through the stationary phase of growth. For example, *H. pylori* expresses two flagellin genes, *flaA* and *flaB*, with transcription of *flaA* highest in late exponential phase, whereas *flaB* transcription peaks in early to mid-exponential phase. Transcription of both genes is negligible in stationary phase [39]. *C. crescentus* flagellum genes are expressed at specific periods of the cell cycle [40], and flagellin transcription in *E. coli* is increased in mid- to late exponential growth phases [41]. Similar to *E. coli*, *R. capsulatus* was found to induce the expression of flagellar genes as cultures progressed from exponential to stationary phase [21]. Perhaps flagellar gene expression during 12 to 20 hours after inoculation of stab-plates is repressed, as in liquid medium cultures in the stationary phase. This would explain the induction of flagellar motility, as evidenced by the broadening of the line of cell density and the appearance of distal colonies, after 28 hours in wild type strain stab-plates.

Purple photosynthetic bacteria exhibit a “scotophobic”, or “*Schreckbewegung*” response in liquid media, which results from a random reorientation due to tumbling when a cell moves from a region of high light intensity to a lower light intensity [42]. The net result is accumulation of cells in regions of high light intensity. The scotophobic response could explain why flagellum-dependent motility in stab-plates illuminated with white light results in a broadening of the line of cell movement. The accumulation of cells due to replication may have contributed to changes in the transmission of light through the culture to individual cells because of self-shading (absorption or scattering of light by cells). Upon induction of flagellar motility, cells moving away from the line of high cell density would experience an increase in light intensity and keep moving in the same direction, whereas cells entering into a shaded region would have induced flagellar tumble reorientations, with a net movement away from the flagellum-independent path of linear motility [42].

### Quorum sensing and *R. capsulatus* solid-substrate motility

In *P. aeruginosa* twitching motility and *M. xanthus* social motility, cells that form a motile mass undergo alignments along the long axis of the cells upon coming into contact with each other to organize into raft-like clusters [43], [44]. It is unclear how *R. capsulatus* cells initiated and maintained the organization of cells into an aggregated mass for movement as shown in Figures 3 through [Fig pone-0019646-g004] 5. However, this pattern of motility occurred without the HSL quorum sensing signal [30] produced by GtaI (Figure 5), which indicates that some other signaling method coordinated the groups of cells that formed satellite clusters. *P. aeruginosa* and *M. xanthus* cells travel up a phosphatidylethanolamine gradient on solid substrates, promoting cell-cell contact to create groups of cells for movement [45], [46]. Chemotaxis has not been demonstrated in *R. capsulatus*, although the genome encodes 18 putative methyl-accepting chemotaxis proteins and a homologue of the *nnrS* nitrate chemotaxis protein of *R. sphaeroides* [29], [47], [48], [49]. Perhaps the organization of cells for flagellum-independent motility is controlled by an uncharacterized chemotactic response between *R. capsulatus* cells.

### The rate and possible mechanisms of *R. capsulatus* flagellum-independent, solid-substrate motility

The rate of flagellum-independent *R. capsulatus* cell movement determined by microscopy was similar to that estimated in macroscopic examinations of time-course stab-plates. The macroscopic time-course results showed that flagellum-independent movement in the plate-medium interface yielded growth at the plate periphery within 12 hours of inoculation (Figure 3). To have reached the plate edge from the site of inoculation within this time, the rate of cell movement had to be at least ∼1 µm/sec (a distance of ∼45 mm traveled in ≤12 hours). The flagellum-deficient *flaA* mutant strain bKSDF cells visualized in the plate-medium interstice by microscopy moved at a rate of ∼1.1 µm/sec (Figure 6). Therefore two independent measurements indicate the same rate, and so the observed microscopic movements of single cells could mediate the collective movement of a cell mass as seen in the time-course stab-plates.

There are several mechanisms for prokaryotic movement across surfaces, including swarming, twitching, sliding, darting, and gliding motility. *R. capsulatus* solid-substrate motility is not flagellum-dependent swarming because *flaA* and *ctrA* mutant strains are still motile [22]. Although *R. capsulatus* contains homologues of genes encoding core components of a type IV pilus [50] potentially involved in twitching motility, knockouts of these genes did not interfere with flagellum-independent movement (data not shown). A sliding motility mechanism also seems unlikely because the sliding movement of individual or groups of cells relative to other cells does not occur [6]. Sliding motility is a passive form of translocation [13], [51] that would not be expected to generate directed movement toward white light (Figure 7). Although little is known about darting motility, individual cells appear to dart in quick random movements [6], [52] unlike that observed in *R. capsulatus* (Video S1). Instead, the flagellum-independent movement of *R. capsulatus* occurred in a progressive manner as an aggregated mass toward white light. However the rate of flagellum-independent movement was within the range of rates observed for gliding motility, which vary from ∼0.02 to 10 µm/sec depending on the composition of the medium and the bacterial species [5]. We speculate that the flagellum-independent movement of *R. capsulatus* is driven by a gliding motility mechanism [53], [54], although our current experimental data are not sufficient to specify the structures or components of this mechanism.

In conclusion, it appears that *R. capsulatus* cells move as individual cells and in organized groups across a solid substrate, independently of the flagellum in what could be a gliding motility mechanism. The coordinated movement is manifested as an aggregated mass of cells that travel toward broad spectrum white light in the absence of GtaI-dependent quorum sensing, at a rate of at least 1 µm/sec on a 1.5% agar surface in a glass-agar interstice.

## Supporting Information

Video S1 Time-lapse microscopy video of an agar plate inoculated with *flaA* mutant strain bKSDF. An image was captured each 0.25 seconds over the course of 150 seconds to record flagellum-independent individual cell movements. Only 460 of the 600 frames are shown from a 115 second interval of cell movement.
(WMV)
